# Abnormal expression of Tim‐3 antigen on peripheral blood T cells is associated with progressive disease in osteosarcoma patients

**DOI:** 10.1002/2211-5463.12079

**Published:** 2016-07-09

**Authors:** Hongliang Liu, Liqiang Zhi, Ning Duan, Pengxiao Su

**Affiliations:** ^1^ Department of Traumatic OsteopathicXi'an Honghui HospitalXi'an Jiaotong University College of MedicineShanxiChina; ^2^ Department of Articular OsteopathicXi'an Honghui HospitalXi'an Jiaotong University College of MedicineShanxiChina; ^3^ Department of SurgeryXi'an Honghui HospitalXi'an Jiaotong University College of MedicineShanxiChina

**Keywords:** diagnosis, osteosarcoma, Tim‐3 protein, T‐lymphocytes

## Abstract

T‐cell immunoglobulin and mucin‐domain‐3‐containing molecule 3 (TIM‐3) plays a pivotal role in immune regulation and has been found in various tumors. However, the prevalence and distribution of Tim‐3 in osteosarcoma (OS) is still unclear. The aim of this study was to investigate the prevalence and distribution of Tim‐3 in OS. Tim‐3 on peripheral T cells from 82 OS patients and 60 healthy controls were examined by flow cytometry. Plasma levels of IL‐2, IFN‐γ, and TNF‐α were measured by ELSIA. Tim‐3 on both CD4^+^ T and CD8^+^ T cells were significantly upregulated in OS patients compared with healthy controls, Tim‐3^+^ CD4^+^ T, and Tim‐3^+^ CD8^+^ T cells were both negatively associated with serum levels of IL‐2 and IFN‐γ and TNF‐α. In addition, Tim‐3 showed similar levels in patients with different tumor sites. Nevertheless, patients with advanced tumor stage, metastasis, and pathological tumor fracture displayed significantly higher Tim‐3 on both CD4^+^ T cells and CD8^+^ T cells than those with early tumor stage, without metastasis and pathological tumor fracture. Moreover, high Tim‐3 on peripheral CD4^+^ T cells or CD8^+^ T were significantly related to poor overall survival (*P* = 0.014, *P* = 0.035, respectively). In conclusion, Tim‐3 may be a potential diagnostic and prognostic biomarker for OS progression.

**Abbreviations**
EMT: epithelial–mesenchymal transition
OS: osteosarcoma
PBMCs: peripheral blood mononuclear cells
Tim‐3: T‐cell immunoglobulin and mucin‐domain‐containing molecule 3

Osteosarcoma (OS) is the most common primary malignant bone tumor. Carcinogenesis and the mechanisms affecting the progression and prognosis of OS involve a multistep process 1. Despite significant advances in surgical techniques and chemotherapeutic treatment, patients with distant metastases usually have poor prognosis 2. Therefore, it is crucial to classify new diagnostic and prognostic molecular biomarkers for predicting the progression of OS and helping targeted therapy.

T‐cell immunoglobulin and mucin‐domain‐containing molecule 3 (Tim‐3), which is mainly expressed on Th1 cells but not on Th2 cells, belongs to TIMs family 3. Tim‐3 inhibits Th1 responses and induces peripheral tolerance through binding to its potential ligand, galectin‐9, or CEACAM1 4, 5, suggesting an inhibitory role of Tim‐3 in immune response. In fact, Tim‐3, along with cytotoxic T lymphocyte antigen 4 (CTLA‐4) and Programmed Death 1 (PD‐1), are recently identified as an immune checkpoint molecules 6.

Recent studies demonstrated a vital biological role of Tim‐3 in a variety of tumors 7. Tim‐3‐expressing CD4^+^ and CD8^+^ T cells are significantly increased in nonsmall‐cell lung cancer patients 8, 9. In addition, Tim‐3 and PD‐1 are coexpressed on tumor‐infiltrating CD8^+^ T cells in mice bearing transplanted tumors, as well as on NY‐ESO‐1‐specific CD8^+^ T cells in patients with advanced melanoma 10, 11. Tim‐3 could also suppress CD4^+^ T cells activation through interleukin‐6‐STAT3 pathway and also facilitate the establishment of lymphoma immune tolerance 12. Therefore, targeting Tim‐3 pathways can converse T‐cell exhaustion and reestablish antitumor immune responses 13, 14.

However, there have been few studies reporting the expression of Tim‐3 in OS. It has been shown that Tim‐3 was restricted in the cytoplasm and the membrane of OS cells 15. Interestingly, some epithelial‐mesenchymal transition (EMT) biomarkers, such as vimentin, Slug, Snail, and Smad, were found coexpressed with Tim‐3 in sarcoma cells, indicating that TIM‐3 may be involved in the pathogenesis of OS 15. In this study, we analyzed Tim‐3 expression on peripheral CD4^+^ and CD8^+^ T cells in OS patients.

## Materials and methods

### Study subjects

This study was approved by the Research Ethics Committee of Xi'an Honghui Hospital, P. R. China. Written informed consent was obtained from all of the patients according to the committee's regulations. Peripheral blood samples were collected from 82 OS patients and 60 healthy controls in Xi'an Honghui Hospital between 2011 and 2014. OS diagnosis was performed with histological examination. OS stage was determined using the Musculoskeletal Tumor Society (MSTS) Staging System. Patients who had undergone any form of preoperative chemotherapy and/or radiation therapy were excluded. All the control subjects were matched with patient population in terms of age and sex. Follow‐up was performed for 56 OS patients from 8 to 56 months (median, 39 months) and ended Apr 10, 2016. Overall survival was defined as the interval between initial surgical operation and death. Selected characteristics of the cases and controls are presented in Table 1.

**Table 1:** General characteristics of the OS patients and healthy controls

Characteristics	Osteosarcoma (*n* = 82) (%)	Control (*n* = 60) (%)	*P* value
Gender			
Male	48 (58.5)	36 (60)	NS
Female	34 (41.5)	24 (40)	
Age			
≤ 20	56 (68.3)	40 (66.7)	NS
> 20	26 (31.7)	20 (33.3)	
Tumor site			
Femur	37 (45.1)		
Tibia	20 (24.4)		
Others^a^	25 (30.5)		
Tumor stage			
I	17 (20.7)		
II	58 (70.7)		
III	7 (8.6)		
Distant metastasis			
Yes	7 (8.6)		
No	75 (91.4)		
Pathological fracture			
Yes	13 (15.8)		
No	69 (84.2)		

NS, no significant (*P* > 0.05). ^a^Others include Humerus (11 patients), pelvic (5 patients), radius (5 patients), and ulna (4 patient).

### Cell preparation and flow cytometry

Peripheral blood mononuclear cells (PBMCs) were isolated from heparinized peripheral blood from the study subjects by Ficoll density gradient centrifugation (Hao Yang, Tianjin, China). The cells were washed and 2 × 10^6^ cells were stained with monoclonal antibodies or isotype‐matched controls for 30 min on ice. Fluorescein isothiocyanate (FITC)‐anti‐human CD4, phycoerythrin (PE)‐ anti‐human Tim‐3, phycoerythrin‐Cyanine 5.5 (PE/Cy5.5)‐anti‐human CD3, and allophycocyanin (APC)‐anti‐human CD8 (all from eBioscience, San Diego, CA, USA) were used for flow cytometric analysis. Data were acquired on a FACS Calibur (BD Bioscience, San Diego, CA, USA) and analyzed using cellquest software (BD Bioscience).

### ELISA

Plasma was obtained after centrifugation at 800 ***g*** for 10 min. The concentrations of interleukin‐2 (IL‐2), interferon‐γ (IFN‐γ) and tumor necrosis factor‐α (TNF‐α) were measured by ELISA according to the manufacturer's instructions (eBioscience). All samples were measured in duplicate.

### Statistical analysis

All data were analyzed using the graphpad prism 5 (GraphPad Software Inc., San Diego, CA, USA). Data were presented as means ± SEM. Differences between the values were determined using student's t‐test. Correlation analysis was evaluated by the Spearman ρ correlation test. Survival curves were plotted using the Kaplan–Meier method, and differences between survival curves were tested using the log‐rank test. Significance was determined as *P < 0.05, **P < 0.01, ***P < 0.001.

## Results

### Clinical characteristics of the study subjects

Selected characteristics of the 82 OS patients and 60 healthy controls are presented in Table 1. Patients and control subjects did not reveal any statistically significance in terms of age (*P* > 0.05) and sex (*P* > 0.05). Of all the 82 patients, 17 (20.7%) were in stage I, 58 (70.7%) were in stage II, 7 (8.6%) were in stage III. A total of 8 (8.6%) patients had distant metastasis, and 13 (15.8%) patients had pathological bone fracture.

### Increased Tim‐3 on peripheral CD4^+^ and CD8^+^ T cells in OS patients

**Figure 1:**
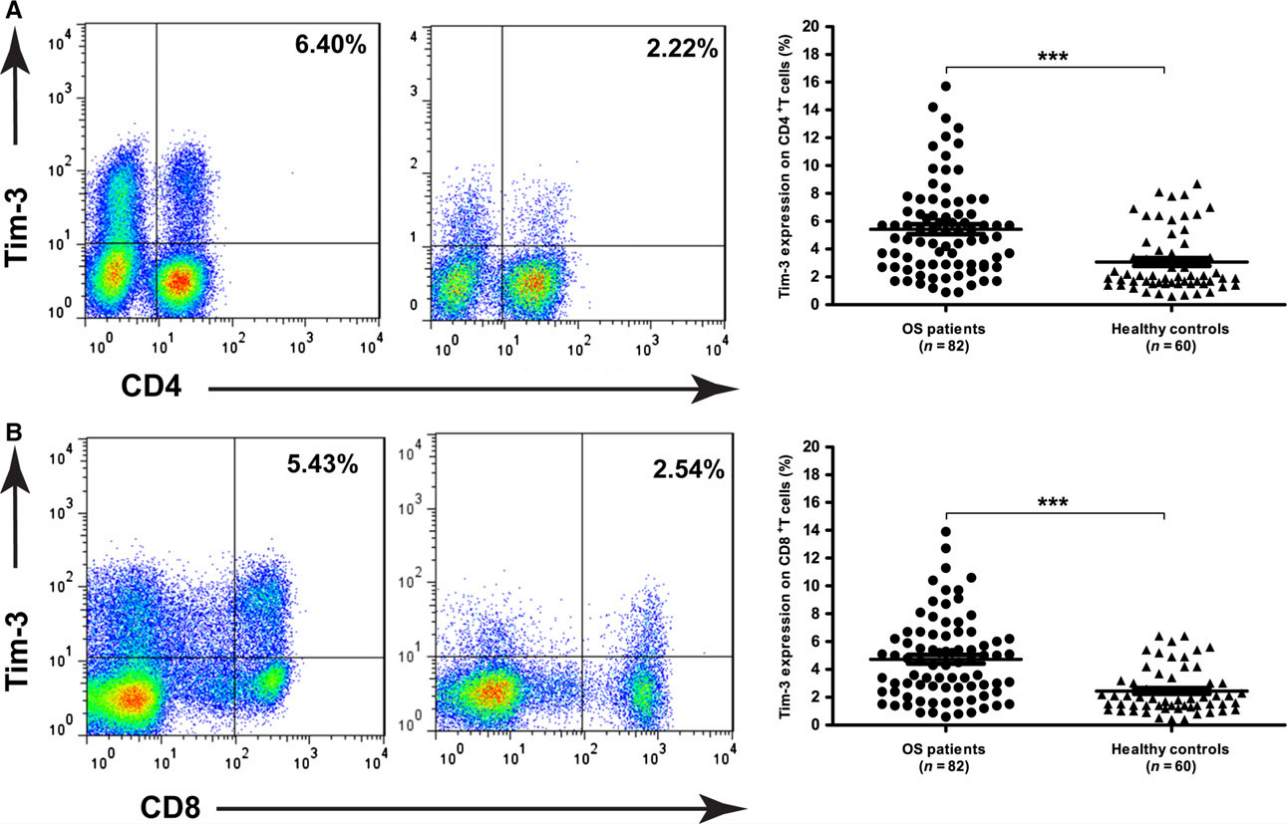
Tim‐3 was significantly increased on peripheral CD4^+^ and CD8^+^ T cell in OS patients (*n* = 82) than in healthy controls (*n* = 60). Data shown are the representative FACS profiles and proportions of Tim‐3 on CD4^+^ T cells (A) and CD8^+^ T cells (B) in patients and controls. Each dot represents one subject. Data are shown as mean ± SEM. ***P < 0.001.

**Figure 2:**
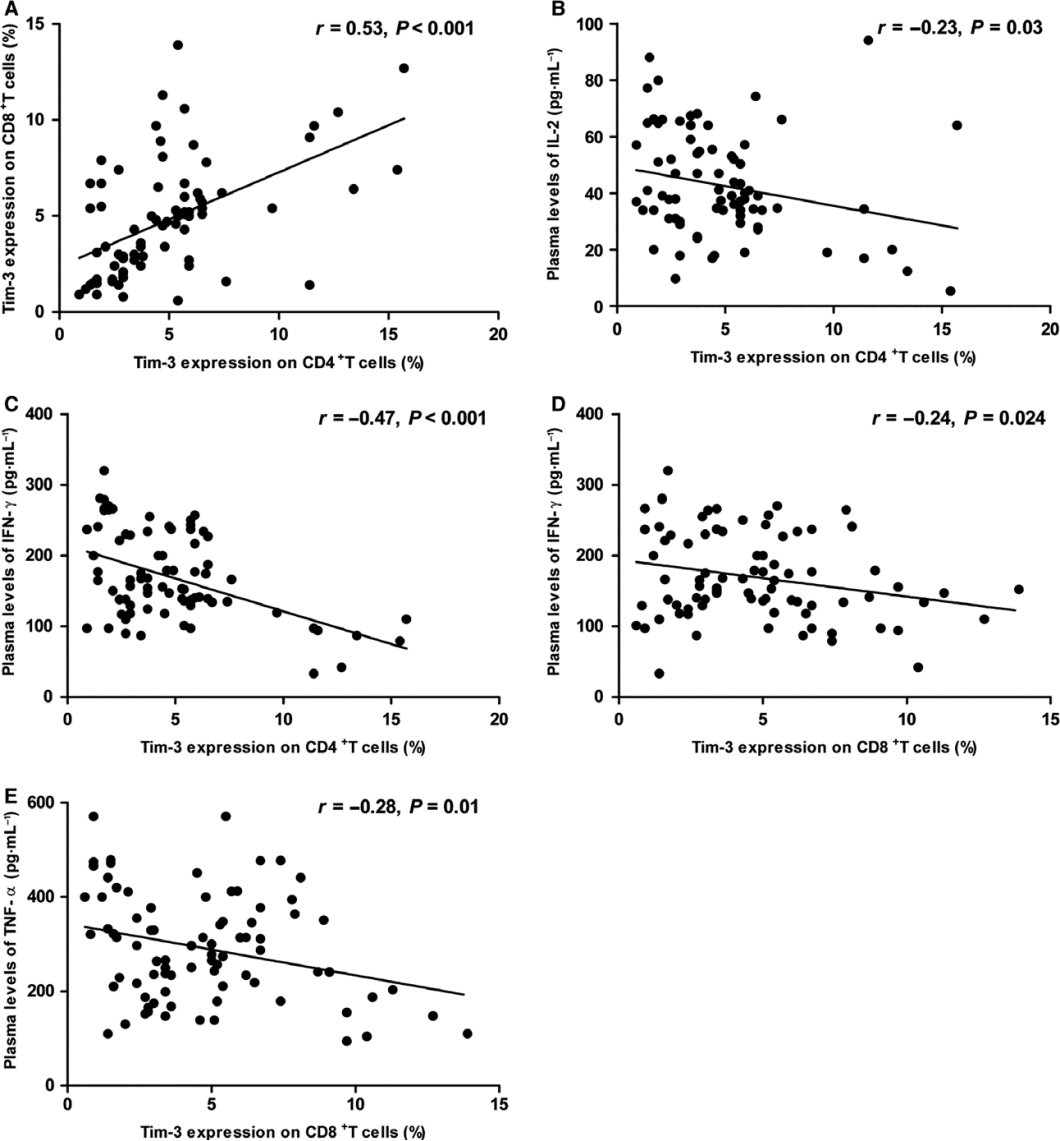
Correlation between Tim‐3 on CD4^+^ and on CD8^+^ T cells in OS patients (A). Correlation between Tim‐3 on CD4^+^ T cells and plasma levels of IL‐2 (B) and IFN‐γ (C). Correlation between Tim‐3 on CD8^+^ T cells and plasma levels of IFN‐γ (D) and TNF‐α (E). A total of 82 OS patients were included. Each dot represents one subject.

To understand the role of Tim‐3 in OS, we first examined the expression patterns of Tim‐3 on peripheral CD4^+^ T cells and CD8^+^ T cells in OS patients (*n* = 82) and healthy controls (*n* = 60). As shown in Fig. 1A, Tim‐3 on CD4^+^ T cells was significantly upregulated in OS patients than that in controls (mean ± SEM: 5.429 ± 0.36% vs. 3.07 ± 0.28%, *P* < 0.0001). Likewise, Tim‐3 on CD8^+^ T cells was also significantly upregulated in patients compared with that of controls (4.737 ± 0.32% vs. 2.458 ± 0.21%, *P* < 0.001) (Fig. 1B). We next analyzed the correlations between Tim‐3 on CD4^+^ T cells and CD8^+^ T cells in OS patients. As shown in Fig. 2A, Tim‐3 on CD4^+^ T cells was positively correlated with Tim‐3 on CD8^+^ T cells in OS patient group (*r* = 0.53, *P* < 0.001), while no correlation between Tim‐3 on CD4^+^ T cells and CD8^+^ T cells in healthy controls was observed (data not shown). We next analyzed the associations between Tim‐3 on T cells and the plasma levels of IL‐2, IFN‐γ, and TNF‐α in OS patient. Data showed that Tim‐3 on CD4^+^ T cells was negatively associated with serum levels of IL‐2 and IFN‐γ (*r* = −0.23, *P* = 0.03; *r* = −0.47, *P* < 0.001, respectively, Fig. 2B,C), and Tim‐3 on CD8^+^ T was adversely correlated with serum levels of IFN‐γ and TNF‐α (*r* = −0.24, *P* = 0.024; *r* = −0.28, *P* = 0.01, respectively, Fig. 2D,E), whereas there was no correlation between Tim‐3 on CD4^+^ T cells and plasma levels of TNF‐α, as well as Tim‐3 on CD8^+^ T cells and plasma levels of IL‐2 (data not shown).

### Similar Tim‐3 in patients with different primary tumor locations

**Figure 3:**
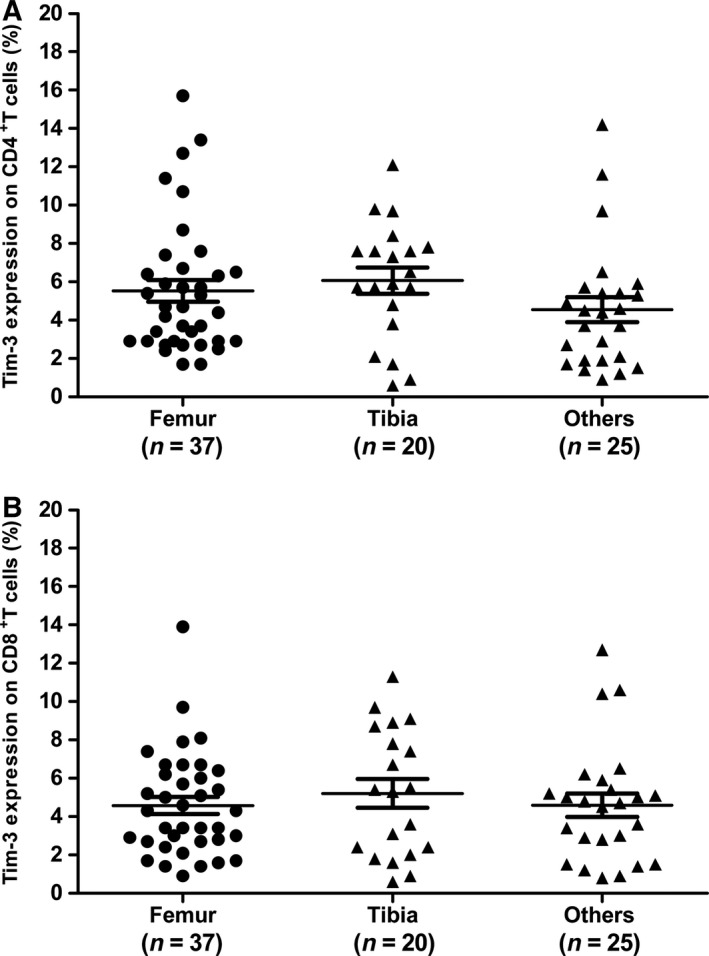
Tim‐3 on CD4^+^ T cells (A) and CD8^+^ T cells (B) in OS patients with different primary tumor sites. Each dot represents one subject. Data are shown as mean ± SEM.

Osteosarcoma frequently arises at the sites of bone growth; most often, it affects the distal end of femur, or proximal end of tibia or humerus. In this study, 37 patients had the primary tumor site at femur, 20 patients at tibia, and 25 patients at other locations (Table 1). We then examined Tim‐3 on peripheral CD4^+^ and CD8^+^ T cells in patients with different primary tumor locations. As shown in Fig. 3, Tim‐3 on both peripheral CD4^+^ T cells and CD8^+^ T cells were similar among patients with different tumor locations, suggesting that primary tumor sites do not have an effect on Tim‐3 expression.

### Increased Tim‐3 in patients with advanced tumor stages

**Figure 4:**
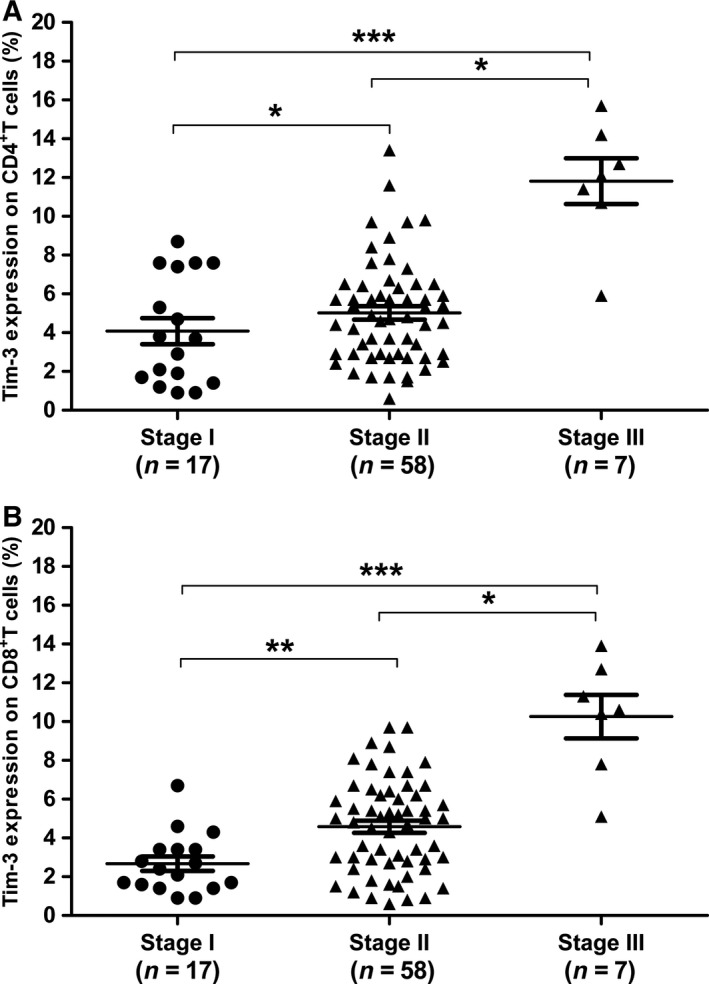
Tim‐3 on CD4^+^ T cells (A) and CD8^+^ T cells (B) in OS patients with different tumor stages. Each dot represents one subject. Data are shown as mean ± SEM. *P < 0.05, **P < 0.01, ***P < 0.001.

Patients with different tumor stages may have diverse prognosis. For example, OS patients with stage I has an excellent prognosis (> 90%) with wide tumor resection, while the overall survival prognosis of stage III patients with lung metastases is only about 30%. We then analyzed Tim‐3 on peripheral CD4^+^ and CD8^+^ T cells in patients with different tumor stages. The results demonstrated that Tim‐3 on CD4^+^ T cells was positively associated with tumor stage. In other words, patients with later‐stage had higher Tim‐3 expression on CD4^+^ T cells (Fig. 4A). Patients with advanced stages of tumor also presented significantly higher Tim‐3 on CD8^+^ T cells than those with primary stages of tumor (Fig. 4B).

### Increased Tim‐3 in patients with tumor metastasis

**Figure 5:**
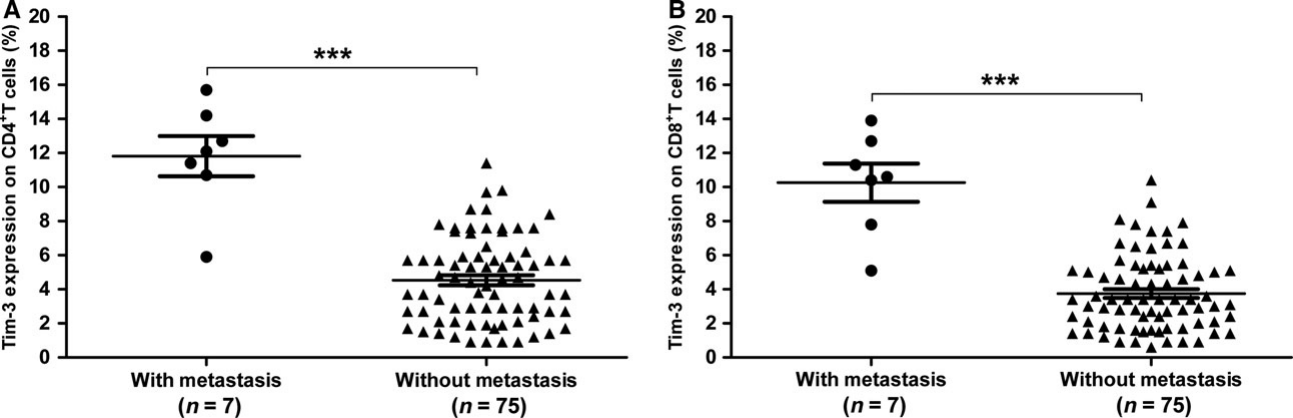
Tim‐3 on CD4^+^ T cells (A) and CD8^+^ T cells (B) in OS patients with different metastatic status. Each dot represents one subject. Data are shown as mean ± SEM. ***P < 0.001.

In this study, patients with metastasis had significantly higher Tim‐3 on CD4^+^ T cells (11.81 ± 1.17% vs. 4.53 ± 0.29%, *P* < 0.001, Fig. 5A), and on CD8^+^ T cells (10.26 ± 1.12% vs. 3.75 ± 0.26%, *P* < 0.001, Fig. 5B) than those without metastasis.

### Increased Tim‐3 in patients with pathological bone fracture

**Figure 6:**
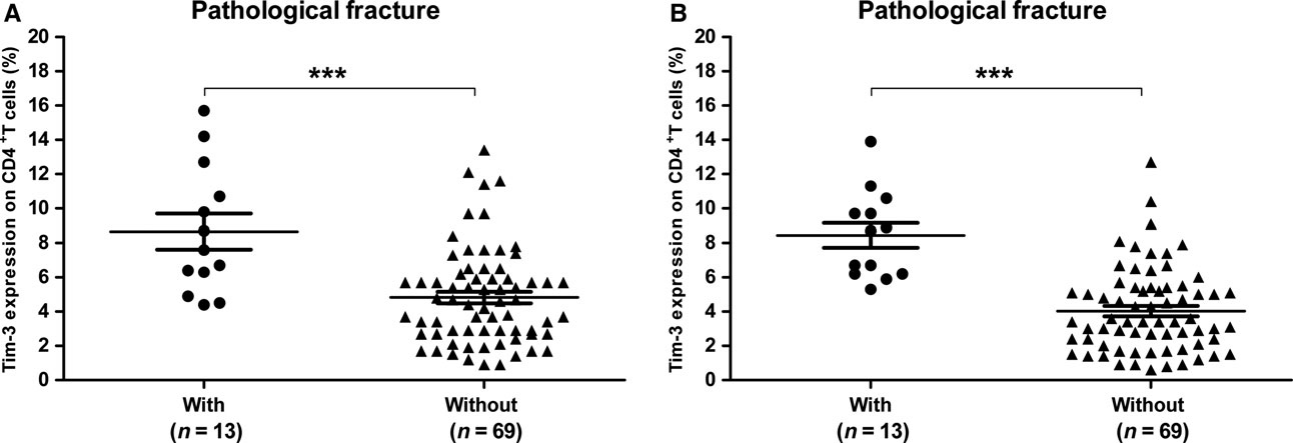
Tim‐3 on CD4^+^ T cells (A) and CD8^+^ T cells (B) in OS patients with different status of pathological bone fracture. Each dot represents one subject. Data are shown as mean ± SEM. ***P < 0.001.

Pathological bone fracture is commonly associated with disease severity in OS patients. In this study, Tim‐3 on CD4^+^ T cells and CD8^+^ T cells significant elevated in patients with pathological fracture compared with those without pathological fracture (8.662 ± 1.04% vs. 4.833 ± 0.34%, *P* < 0.001, Fig. 6A; 8.446 ± 0.71% vs. 4.04 ± 0.29%, *P* < 0.001, Fig. 6B).

### Correlation between Tim‐3 and prognosis in OS patients

**Figure 7:**
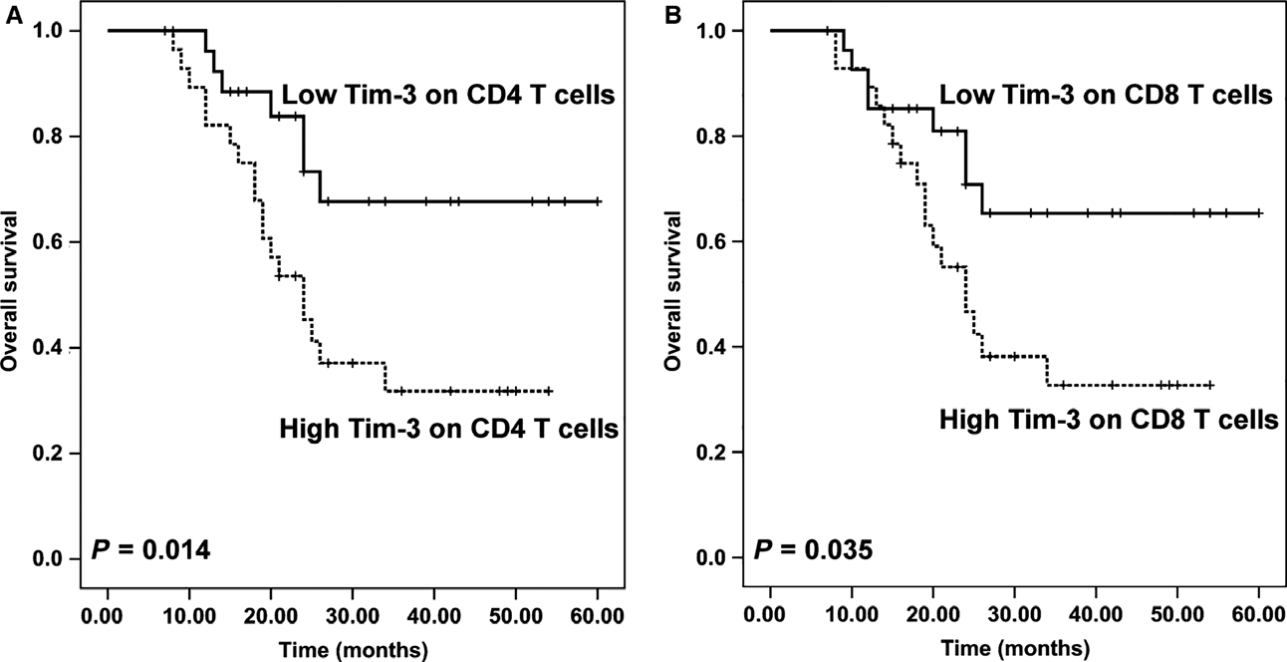
Overall survival of patients with high or low Tim‐3 on peripheral CD4^+^ T cells (A) or CD8^+^ T cells (B). The median value of Tim‐3 levels on peripheral CD4^+^ T cells or CD8^+^ T cells was defined as the cutoff value to divide patients into groups with high and low Tim‐3 levels (*n* = 28 per group). Survival curves were plotted using Kaplan–Meier method and analyzed using the log‐rank test.

The prognostic value of Tim‐3 in human OS was further explored by Kaplan–Meier analysis and the log‐rank test. We used median value as the cutoff value to divide patients into groups with high and low Tim‐3 levels (*n* = 28 per group). As shown in Fig. 7A,B, Overall survival of patients with a high Tim‐3 on peripheral CD4^+^ T cells was significantly lower than survival of those with a low Tim‐3 (*P* = 0.014). In addition, OS patients with high Tim‐3 on peripheral CD8^+^ T cells had shorter overall survival (*P* = 0.035).

## Discussion

To date, there have been limited studies on the role of the Tim‐3 in OS. A recent study by Shang *et al*. 15 found that Tim‐3 expression in human OS tissues was association with the expression of EMT‐specific biomarkers, including vimentin, Slug, Snail, and Smad, suggest that TIM‐3 elicits tumor cells to acquire aggressive EMT features and may be involved in OS pathogenesis. Tim‐3 was originally recognized as Th1‐specific markers, and has been found on CD8^+^ T cells, antigen presenting cell (APCs), NK cells and NKT cells, melanoma, gastric cancer and lung cancer cells 7, 8, 16, 17. However, the prevalence and distribution of Tim‐3 on peripheral CD4^+^ and CD8^+^ T cells in OS were not reported.

Here, to our knowledge for the first time, we demonstrated that Tim‐3 was significantly upregulated on peripheral CD4^+^ and CD8^+^ T cells in OS patients. Moreover, Tim‐3 on CD4^+^ T cells was positively correlated with level of Tim‐3 on CD8^+^ T cells in OS patient, suggesting that the expression of Tim‐3 was not cell specific in OS. In addition, Tim‐3 on these cells in patients with advanced tumor stages was higher than those with early stages. More importantly, we found that Tim‐3 expression on these cells in patients with distant metastasis and pathological tumor fracture was significantly higher than those without. However, patients with different tumor sits showed comparable Tim‐3 levels. These data suggest that Tim‐3 may be greatly involved in the pathogenesis of OS and could be a valuable biomarker for OS. In accordance with our results, Song *et al*. reported that Tim‐3 was significantly increased in both CD4^+^ and CD8^+^ T cells in glioma patients than in controls. Patients with a higher tumor grade shown elevated Tim‐3 on CD8^+^ T cells compared with those with a lower tumor grade 18. Xiao *et al*. 19 found that Tim‐3 was significantly elevated on both CD4^+^ and CD8^+^ T cells in diffuse large B cell lymphoma patients than in healthy controls. Consistent with this observation, Tim‐3 may be an attractive candidate for the treatment of cancer.

Tim‐3 is a co‐inhibitory molecular that negatively regulates T‐cell function, high Tim‐3 expression on antigen specific CD8^+^ T cells is associated with T‐cell exhaustion, which impairs the effector functions of CD8^+^ T cells, including the capacity to proliferation, the ability to produce effector cytokines such as IL‐2, TNF‐α, and IFN‐γ 11, 20. Numerous studies have shown that Tim‐3 dysregulation is closely correlated with cancer progression 17, 21, 22, 23. Very recently, Han *et al*. 24, found that Tim‐3 was up‐regulated in peripheral blood and tumor‐infiltrating T cells in OS patients, and coexpressed with PD‐1. These TIM‐3^+^ T cells presented reduced proliferation and proinflammatory cytokine secretion. Accordingly, our results showed that Tim‐3 on CD4^+^ T cells was negatively correlated with plasma levels of IL‐2 and IFN‐γ, and Tim‐3 on CD8^+^ T cells was adversely correlated with plasma levels of IFN‐γ and TNF‐α, suggesting that peripheral T cells were highly exhausted in OS, and this may be closely related to the poor disease progression of OS patients. As expected, our results showed that high Tim‐3 on CD4^+^ T and CD8^+^ T were both correlated with shorter overall survival of OS patients, indicating that Tim‐3 on peripheral blood T cells may be a novel prognosis predictor and therapeutic target of the patients with OS.

The mechanisms for regulating Tim‐3 expression on immune cells and tumor cells are not yet fully understood. Galectin‐9 (Gal‐9) was described as a binding receptor that mediates T‐cell inhibitory effects of Tim‐3. High expression of Gal‐9 was found in patients with gastric cancer, breast cancer, hepatocellular carcinoma, and malignant melanoma 17, 25, 26, 27. Recently, carcinoembryonic antigen cell adhesion molecule 1 (CEACAM1), another well‐known molecule expressed on activated T cells and involved in T‐cell inhibition, was identified as a heterophilic ligand for Tim‐3 that is required for its ability to mediate T‐cell inhibition, and this interaction has a crucial role in regulating autoimmunity and anti‐tumor immunity 4. Previous study showed elevated expression of CEACAM‐1 in NSCLC and high CEACAM‐1 expression was associated with an increased angiogenic activity 28. Further studies are needed to investigate the prevalence and distribution of CEACAM‐1 on immune cells and other tumor cells, and the interaction between Tim‐3 and CEACAM‐1 in tumor development.

In summary, our study identified the elevated Tim‐3 on CD4^+^ T and CD8^+^ T cells from peripheral blood in OS patients, high Tim‐3 levels were positively correlated with tumor stages, metastasis, pathological tumor fracture, as well as poor prognosis. Our data suggest that Tim‐3 maybe a potential diagnostic marker for the progression of OS and might be as a therapeutic target for the treatment of this disease.

## Author contributions

HLL and PXS conceived and designed the experiments. HLL and LQZ performed the experiments. HLL and PXS analyzed the data. ND contributed materials/analysis tools. HLL and PXS wrote the manuscript.
